# Supplementary figures and images for: Innovation indicators based on firm websites—Which website characteristics predict firm-level innovation activity?
Source: PLoS One. 2021 Apr 5;16(4):e0249583. doi: 10.1371/journal.pone.0249583 (PMC8021193; doi:10.1371/journal.pone.0249583)

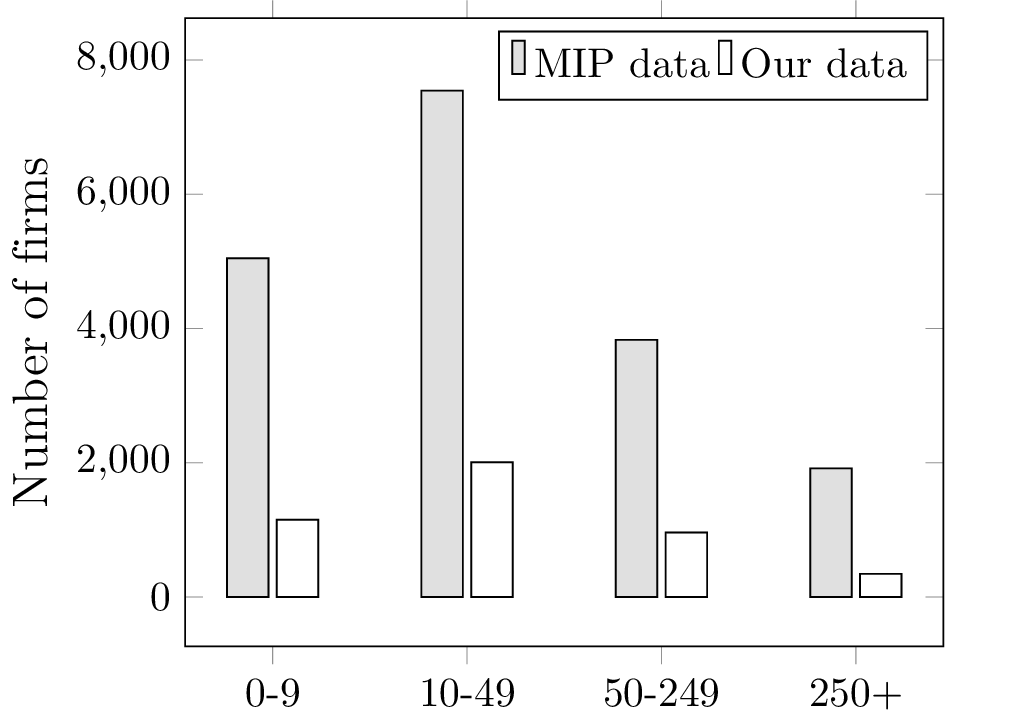

Supplement: S1 Fig — Firm size distribution for the estimation and the full MIP 2019 sample. (TIF) [file pone.0249583.s001.tif]

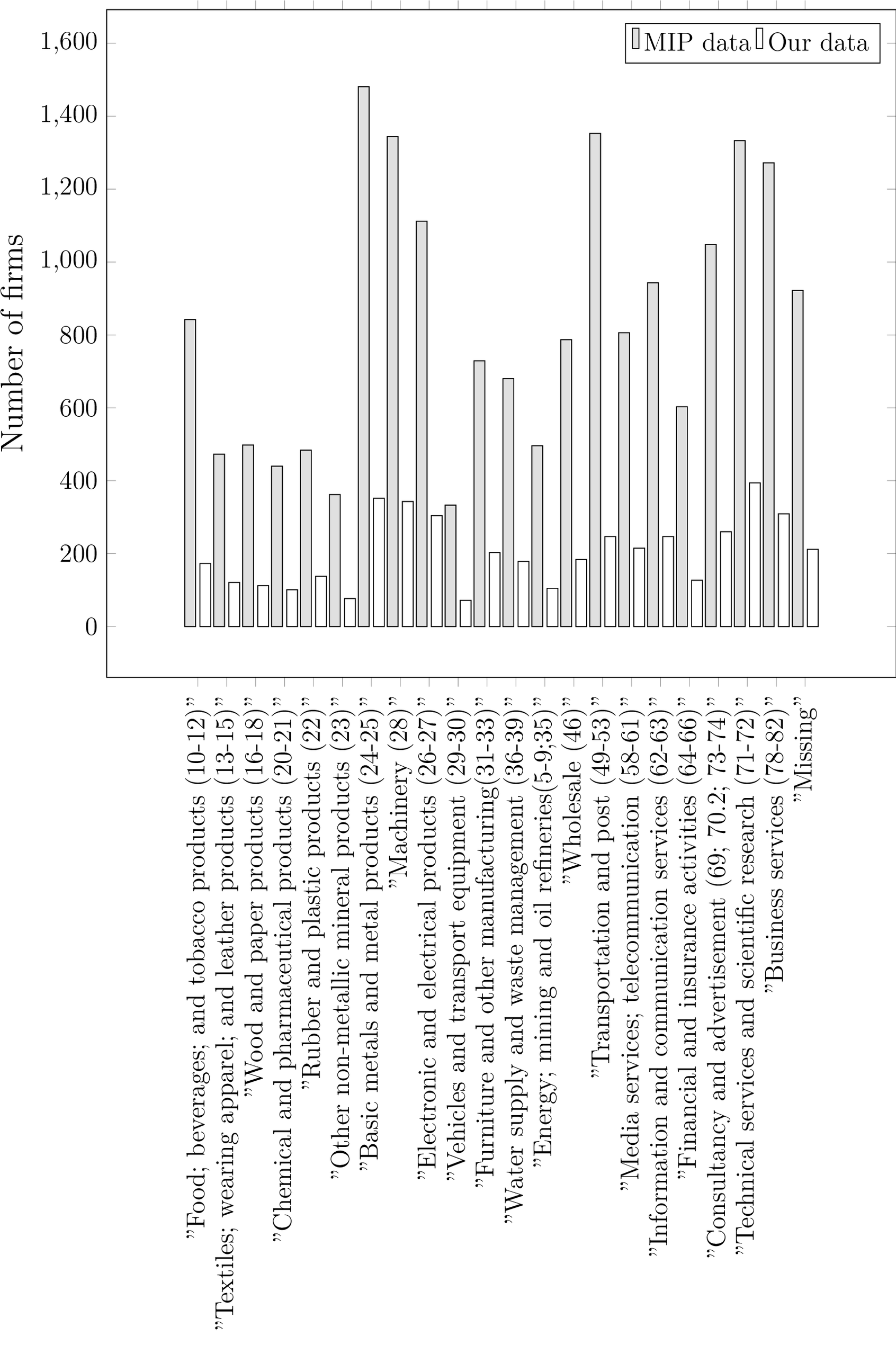

Supplement: S2 Fig — Firm distribution by sector for the estimation sample and the full MIP 2019 data set. (TIF) [file pone.0249583.s002.tif]

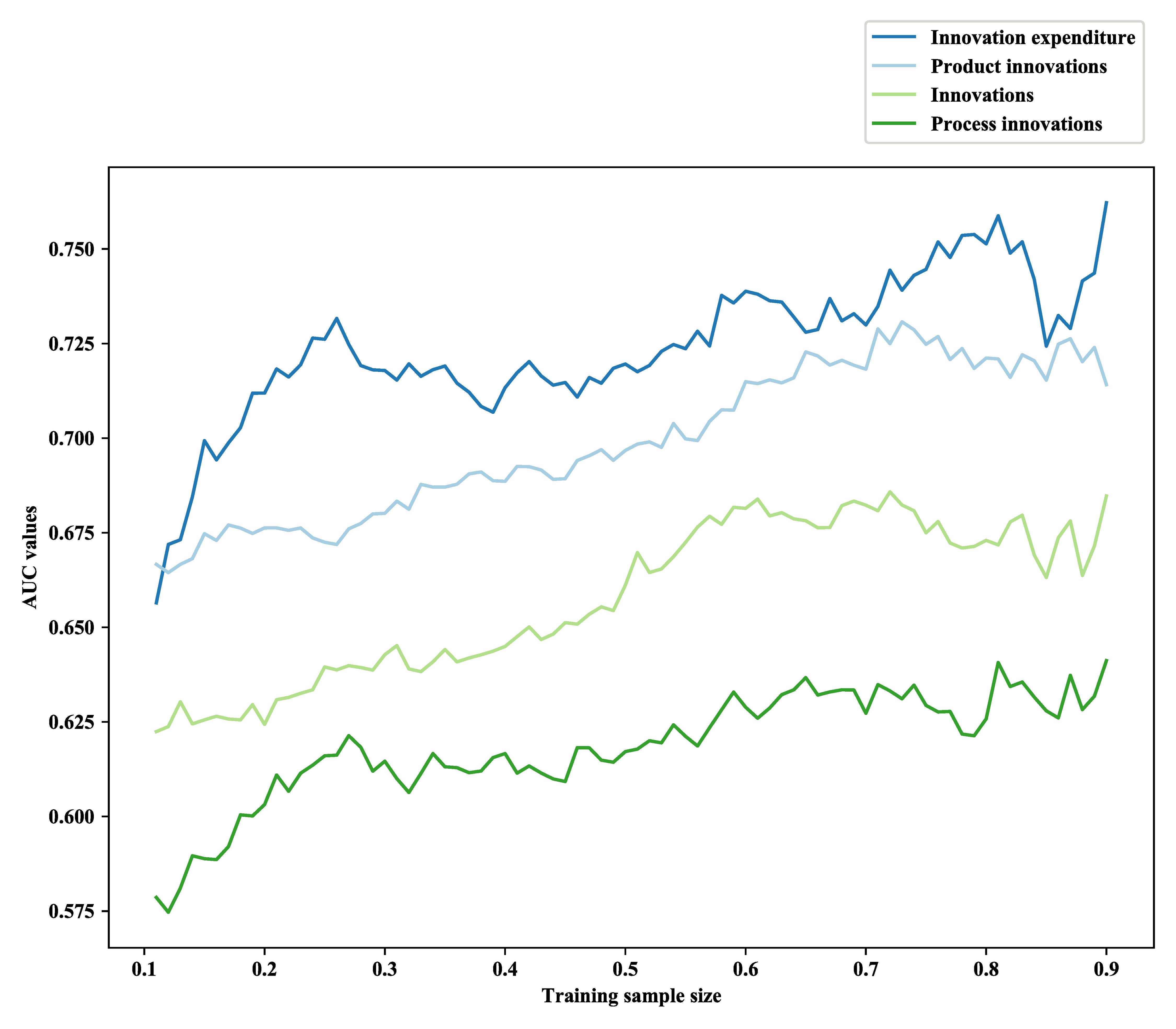

Supplement: S3 Fig — Line plot that illustrates for each indicator how AUC values of the ‘all’ feature model increase if the train/test split changes from (0.1/0.9) to (0.9/0.1) in steps of 0.01. (TIF) [file pone.0249583.s003.tif]
