# Supplementary material for: Innovation indicators based on firm websites—Which website characteristics predict firm-level innovation activity?
Source: PLoS One. 2021 Apr 5;16(4):e0249583. doi: 10.1371/journal.pone.0249583 (PMC8021193; doi:10.1371/journal.pone.0249583)
Supplement: S1 Table — Table with top 100 most relevant features for product innovators, process innovators, innovators and innovation expenditure. (PDF) [file pone.0249583.s004.pdf]

S1 Table: Most relevant features for each ‘all’ feature model 1

Model	Top 100 most relevant features
Product innovators	'LDA topic 35', 'english_language', 'word: system', 'text_length', 'LDA topic 134', 'nr_subpages', 'word: software', 'LDA topic 65', 'word: to develop (transl.)', 'word: application (transl.)', 'LDA topic 105', 'word: test', 'LDA topic 7', 'word: product (transl.)', 'incoming_links', 'word: worldwide (transl.)', 'word: innovative (transl.)', 'LDA topic 98', 'domain_purchase_year_proxy', 'word: version', 'word: innovative', 'LDA topic 41', 'LDA topic 20', 'word: technology (transl.)', 'share_numbers', 'word: sensor', 'LDA topic 127', 'social_media', 'flesch_score', 'word: development (transl.)', 'emerging_tech', 'LDA topic 34', 'word: technology', 'LDA topic 38', 'LDA topic 96', 'LDA topic 75', 'LDA topic 46', 'pop_score', 'LDA topic 39', 'word: automatic (transl.)', 'LDA topic 101', 'LDA topic 70', 'LDA topic 78', 'LDA topic 84', 'LDA topic 128', 'outgoing_links', 'LDA topic 148', 'LDA topic 97', 'word: to optimize (transl.)', 'word: software development (transl.)', 'word: application (transl.)', 'LDA topic 119', 'LDA topic 36', 'word: component (transl.)', 'LDA topic 69', 'load_time', 'LDA topic 52', 'LDA topic 56', 'LDA topic 60', 'LDA topic 143', 'word: digital', 'LDA topic 8', 'LDA topic 113', 'LDA topic 120', 'word: complex (transl.)', 'LDA topic 53', 'LDA topic 138', 'LDA topic 144', 'LDA topic 51', 'LDA topic 15', 'LDA topic 19', 'word: support', 'LDA topic 103', 'LDA topic 106', 'word: user (transl.)', 'LDA topic 57', 'LDA topic 107', 'LDA topic 49', 'LDA topic 104', 'word: deployment (transl.)', 'LDA topic 5', 'LDA topic 111', 'word: interfaces (transl.)', 'LDA topic 85', 'LDA topic 61', 'LDA topic 114', 'LDA topic 43', 'LDA topic 45', 'LDA topic 26', 'LDA topic 132', 'LDA topic 16', 'word: production (transl.)', 'LDA topic 125', 'LDA topic 146', 'word: year (transl.)', 'LDA topic 140', 'LDA topic 91', 'word: integrate (transl.)', 'LDA topic 79', 'word: special (transl.)'
	transl: Translated from German to English language

2

Model	Top 100 most relevant features
Process innovators	'text_length', 'LDA topic 98', 'english_language', 'social_media', 'LDA topic 41', 'flesch_score', 'incoming_links', 'LDA topic 7', 'LDA topic 75', 'word: worldwide (transl.)', 'outgoing_links', 'nr_subpages', 'LDA topic 84', 'word: product (transl.)', 'word: system', 'LDA topic 65', 'LDA topic 20', 'LDA topic 57', 'LDA topic 53', 'share_numbers', 'LDA topic 106', 'LDA topic 148', 'LDA topic 104', 'load_time', 'LDA topic 99', 'LDA topic 122', 'LDA topic 140', 'word: technology (transl.)', 'pop_score', 'word: to develop (transl.)', 'LDA topic 35', 'LDA topic 31', 'LDA topic 127', 'LDA topic 12', 'word: ISO', 'LDA topic 39', 'LDA topic 121', 'LDA topic 32', 'LDA topic 36', 'word: innovative (transl.)', 'LDA topic 2', 'LDA topic 100', 'LDA topic 6', 'LDA topic 13', 'LDA topic 120', 'word: standard', 'word: successful (transl.)', 'LDA topic 43', 'LDA topic 103', 'LDA topic 60', 'LDA topic 64', 'LDA topic 96', 'LDA topic 23', 'LDA topic 133', 'LDA topic 93', 'LDA topic 78', 'LDA topic 40', 'LDA topic 146', 'LDA topic 74', 'LDA topic 101', 'LDA topic 97', 'word: to start (transl.)', 'word: international', 'LDA topic 147', 'LDA topic 86', 'LDA topic 73', 'LDA topic 144', 'LDA topic 14', 'LDA topic 46', 'word: partner', 'LDA topic 19', 'LDA topic 68', 'word: team', 'LDA topic 30', 'LDA topic 141', 'LDA topic 123', 'LDA topic 111', 'LDA topic 34', 'LDA topic 134', 'word: application (transl.)', 'LDA topic 22', 'word: as well as (transl.)', 'LDA topic 0', 'LDA topic 24', 'LDA topic 113', 'LDA topic 88', 'LDA topic 105', 'LDA topic 8', 'LDA topic 94', 'LDA topic 44', 'LDA topic 79', 'LDA topic 114', 'LDA topic 5', 'LDA topic 126', 'LDA topic 83', 'LDA topic 45', 'LDA topic 129', 'LDA topic 56', 'LDA topic 117', 'LDA topic 145'
	transl: Translated from German to English language

3

Model	Top 100 most relevant features	
Innovators	'text_length', 'english_language', 'LDA topic 98', 'nr_subpages', 'word: system', 'word: to develop (transl.)', 'LDA topic 65', 'LDA topic 35', 'word: worldwide (transl.)', 'word: innovative (transl.)', 'LDA topic 84', 'LDA topic 134', 'LDA topic 41', 'LDA topic 20', 'word: product(transl.)', 'LDA topic 7', 'LDA topic 31', 'social_media', 'flesch_score', 'domain_purchase_year_proxy', 'word: development (transl.)', 'word: application (transl.)', 'incoming_links', 'LDA topic 78', 'outgoing_links', 'LDA topic 96', 'LDA topic 75', 'word: successful (transl.)', 'LDA topic 103', 'word: complex (transl.)', 'LDA topic 101', 'LDA topic 100', 'LDA topic 140', 'share_numbers', 'LDA topic 5', 'LDA topic 105', 'LDA topic 122', 'LDA topic 0', 'LDA topic 56', 'LDA topic 114', 'load_time', 'LDA topic 127', 'LDA topic 50', 'LDA topic 6', 'LDA topic 53', 'LDA topic 69', 'LDA topic 94', 'LDA topic 51', 'LDA topic 46', 'LDA topic 120', 'pop_score', 'LDA topic 102', 'LDA topic 90', 'LDA topic 113', 'word: to offer (transl.)', 'LDA topic 121', 'LDA topic 36', 'LDA topic 52', 'LDA topic 32', 'LDA topic 19', 'LDA topic 89', 'word: experience (transl.)', 'LDA topic 2', 'LDA topic 60', 'LDA topic 142', 'word: innovative', 'LDA topic 43', 'LDA topic 23', 'LDA topic 87', 'LDA topic 28', 'LDA topic 39', 'LDA topic 148', 'LDA topic 133', 'LDA topic 106', 'LDA topic 11', 'LDA topic 34', 'LDA topic 82', 'LDA topic 37', 'LDA topic 13', 'LDA topic 86', 'word: as well as (transl.)', 'LDA topic 61', 'LDA topic 33', 'LDA topic 12', 'LDA topic 126', 'word: high (transl.)', 'LDA topic 22', 'LDA topic 71', 'LDA topic 85', 'LDA topic 138', 'LDA topic 144', 'LDA topic 117', 'LDA topic 83', 'LDA topic 16', 'word: deployment (transl.)', 'LDA topic 136', 'LDA topic 147', 'LDA topic 123', 'LDA topic 64', 'LDA topic 68'	4
	transl: Translated from German to English language	

Model	Top 100 most relevant features	
Innovation expend.	'english_language', 'LDA topic 98', 'text_length', 'nr_subpages', 'word: system', 'word: development (transl.)', 'word: to develop (transl.)', 'word: technology', 'LDA topic 134', 'word: innovative (transl.)', 'word: innovation', 'incoming_links', 'word: international', 'LDA topic 148', 'LDA topic 105', 'word: product (transl.)', 'word: application (transl.)', 'word: research (transl.)', 'word: worldwide (transl.)', 'LDA topic 84', 'LDA topic 7', 'domain_purchase_year_proxy', 'LDA topic 36', 'LDA topic 106', 'outgoing_links', 'LDA topic 35', 'flesch_score', 'LDA topic 28', 'LDA topic 5', 'LDA topic 20', 'LDA topic 65', 'load_time', 'LDA topic 100', 'word: innovative', 'LDA topic 39', 'LDA topic 125', 'share_numbers', 'LDA topic 41', 'LDA topic 120', 'LDA topic 73', 'LDA topic 1', 'integration', 'pop_score', 'LDA topic 82', 'LDA topic 13', 'social_media', 'emerging_tech', 'LDA topic 104', 'LDA topic 57', 'LDA topic 6', 'LDA topic 53', 'LDA topic 109', 'LDA topic 26', 'LDA topic 75', 'word: high', 'LDA topic 34', 'LDA topic 32', 'LDA topic 89', 'LDA topic 49', 'LDA topic 140', 'LDA topic 81', 'word: workshop', 'LDA topic 83', 'LDA topic 113', 'word: management', 'LDA topic 22', 'LDA topic 59', 'LDA topic 56', 'LDA topic 31', 'LDA topic 67', 'LDA topic 24', 'LDA topic 0', 'LDA topic 79', 'LDA topic 68', 'LDA topic 102', 'LDA topic 61', 'LDA topic 3', 'LDA topic 138', 'LDA topic 44', 'LDA topic 40', 'LDA topic 128', 'LDA topic 146', 'LDA topic 141', 'word: to optimize', 'LDA topic 70', 'LDA topic 78', 'LDA topic 132', 'LDA topic 95', 'word: process (transl.)', 'LDA topic 80', 'LDA topic 127', 'LDA topic 60', 'LDA topic 93', 'LDA topic 133', 'LDA topic 114', 'LDA topic 46', 'word: high', 'word: as well as', 'LDA topic 96', 'LDA topic 8'	5
	transl: Translated from German to English language	
