# Supplementary material for: Innovation indicators based on firm websites—Which website characteristics predict firm-level innovation activity?
Source: PLoS One. 2021 Apr 5;16(4):e0249583. doi: 10.1371/journal.pone.0249583 (PMC8021193; doi:10.1371/journal.pone.0249583)
Supplement: S2 Table — Details on used software packages. (PDF) [file pone.0249583.s005.pdf]

S2 Table: Details on used software packages

Software	Version
python	3.6.8
igraph	0.1.11
json	2.0.9
langdetect	1.0.7
networkx	2.2
nltk	3.4
numpy	1.16.2
mechanize	0.4.3
pandas	1.1.5
ReadabilityCalculator	0.2.37
regex	2018.1.10
requests	2.21.0
scikit-learn	0.21.2
spacy	2.0.18
