# Supplementary material for: Innovation indicators based on firm websites—Which website characteristics predict firm-level innovation activity?
Source: PLoS One. 2021 Apr 5;16(4):e0249583. doi: 10.1371/journal.pone.0249583 (PMC8021193; doi:10.1371/journal.pone.0249583)
Supplement: S3 Table — Details on hyperparameter combinations of fitted models. (PDF) [file pone.0249583.s006.pdf]

S3 Table: Details on learned hyperparameters

Table 1. Learned hyperparameters for Random Forest models using different feature sets and target variables.

Feature sets			Number of trees	Max. depth	Min. impurity decrease
Text	Meta	Network			
Product innovators					
x			1000	50	0.001
	x		1000	50	0.001
		x	1500	50	0.001
x	x	x	1500	100	0.001
Process innovators					
x			1000	50	0.001
	x		1500	50	0.01
		x	1000	50	0.001
x	x	x	1500	50	0.001
Innovators					
x			1500	50	0.001
	x		1000	50	0.001
		x	500	50	0.001
x	x	x	1000	50	0.001
Innovation expenditures					
x			1500	100	0.001
	x		1000	50	0.01
		x	1000	50	0.01
x	x	x	1000	50	0.001

Source: MIP 2019 and web-scraped data; Own calculations.
