# Supplementary material for: Innovation indicators based on firm websites—Which website characteristics predict firm-level innovation activity?
Source: PLoS One. 2021 Apr 5;16(4):e0249583. doi: 10.1371/journal.pone.0249583 (PMC8021193; doi:10.1371/journal.pone.0249583)
Supplement: S1 Text — Full list of utilized technological terms in German and English language. (PDF) [file pone.0249583.s007.pdf]

S1 Text: List of emerging technology terms used in the conducted keyword search

English terms: Agricultural robot, closed ecological systems, cultured meat, precision agriculture, vertical farming, micro air vehicle, neural-sensing headset, four-dimensional printing, arcology, aerogel, bioplastic, conductive polymers, cryogenic treatment, fullerene, graphene, lab-on-a-chip, magnetorheological fluid, metamaterials, metal foam, multi-function structures, nanomaterials, carbon nanotube, quantum dots, superalloy, synthetic diamond, translucent concrete, 3D displays, ferroelectric liquid crystal display, holography, interferometric modulator display, laser video displays, OLED displays, micro LED displays, telescopic pixel display, time-multiplexed optical shutter, volumetric display, biometrics, digital scent technology, electronic nose, e-textiles, flexible electronics, memristor, molecular electronics, nano electro mechanical systems, spintronics, thermal copper pillar bump, three-dimensional integrated circuit, concentrated solar power, electric double-layer capacitor, flywheel energy storage, grid energy storage, home fuel cell, lithium iron phosphor battery, lithium-sulfur battery, magnesium battery, nanowire battery, ocean thermal energy conversion, smart grid, vortex engine, wireless energy transfer, zero-energy building, computer-generated imagery, virtual reality, ultra-high-definition television, 5G cellular communications, artificial general intelligence, augmented reality, blockchain, carbon nanotube field-effect transistor, civic technology, cryptocurrency, exascale computing, gesture recognition, internet of things, emerging memory technologies, emerging magnetic data storage technologies, fourth generation optical discs, holographic data storage, general purpose computing on graphics processing units, exocortex, machine translation, machine vision, mobile collaboration, nano radio, optical computing, quantum computing, quantum cryptography, radio-frequency identification, semantic web, smart speaker, software-defined radio, speech recognition, subvocal recognition, hybrid forensics, body implants, prosthesis, cryonics, de-extinction, genetic engineering of organisms and viruses, suspended animation, artificial hibernation, immunotherapy/oncology, nano medicines, nano sensors, oncolytic viruses, personalized medicine, whole genome sequencing, robotic surgery, stem cell treatments, synthetic biology, synthetic genomics, tissue engineering, tricorder, brain-computer interface, neuro informatics, electro encephalography, neuro prosthetics, caseless ammunition, directed energy weapon, electro laser, electromagnetic weapons, electrothermal-chemical technology, green bullet, laser weapon, particle beam weapon, sonic weapon, stealth technology, vortex ring gun, wireless long-range electric shock weapon, artificial gravity, stasis chamber, inflatable space habitat, miniaturized satellite, android, gynoid, nanorobotics, powered exoskeleton, self-reconfiguring modular robot, unmanned vehicle, airless tire, alternative fuel vehicle, electro hydrodynamic propulsion, flying car, fusion rocket, hoverbike, jetpack, backpack helicopter, maglev train, vactrain, magnetic levitation, mass driver, personal rapid transit, physical internet, scooter-sharing system, propellant depot, reusable launch system, space elevator, spaceplane, supersonic transport, vehicular communication systems.

German terms: Agrarroboter, geschlossenes ökologisches System, Zuchtfleisch, Präzisionslandwirtschaft, vertikale Landwirtschaft, Mikro-Luftfahrzeug, neuronales Headset, vierdimensionales Drucken, Arkologie, Aerogel, Bio-Kunststoff, leitfähige Polymere, kryogene Behandlung, Fulleren, Graphen, Labor auf einem Chip, magnetorheologische Flüssigkeit, Metamaterialien, Metallschaum, Multifunktionsstrukturen, Nanomaterialien, Kohlenstoffnanoröhre, Quantenpunkte, Superlegierung, synthetischer Diamant, durchsichtiger Beton, 3D-Display, ferroelektrische Flüssigkristallanzeige, Holographie, interferometrische Modulatoranzeige, Laser-Video-Display, OLED Display, Mikro-LED Display, Teleskop-Pixelanzeige,

zeitgemultiplexter optischer Verschluss, volumetrische Anzeige, Biometrie, digitale Dufttechnologie, elektronische Nase, E-Textil, flexible Elektronik, Memoristor, molekulare Elektronik, nanoelektromechanisches System, Spintronik, Thermo-Kupfer-Säulen-Stoß, dreidimensionale integrierte Schaltung, konzentrierte Solarenergie, elektrischer Doppelschicht-Kondensator, Schwungradspeicherung, Speicherung von Netzenergie, Heim-Brennstoffzelle, Lithium-Eisen-Phosphor-Batterie, Lithium-Schwefel-Batterie, Magnesium-Batterie, Nanodraht-Batterie, Ozean-Thermische Energieumwandlung, intelligentes Netz, Vortex-Motor, drahtlose Energie-Übertragung, Nullenergiehaus, computergeneriertes Bild, virtuelle Realität, hochauflösendes Fernsehen, 5G zellulare Kommunikation, künstliche Intelligenz, erweiterte Realität, Blockchain, Kohlenstoffnanoröhren-Feldeffekttransistor, zivile Technik, Kryptowährung, Exascale-Computing, Gestenerkennung, Internet der Dinge, neue Speichertechnologie, neue magnetische Speichertechnologie, optische Platten der vierten Generation, holografischer Speicher, allgemeines Rechnen auf Grafikprozessoren, Exokortex, maschinelle Übersetzung, maschinelles Sehen, mobile Zusammenarbeit, Nano-Funk, optische Datenverarbeitung, Quantencomputer, Quantenkryptographie, Radiofrequenz-Identifikation, semantisches Web, intelligenter Lautsprecher, Software-definiertes Radio, Spracherkennung, subvokale Erkennung, Hybrid-Forensik, Körperimplantat, Kryonik, Wiederbelebung ausgestorbener Tierarten, Gentechnik, verzögerte Reanimation, künstlicher Winterschlaf, Immuntherapie/-onkologie, Nanomedizin, Nanosensoren, onkolytische Viren, individualisierte Medizin, whole genome sequencing, Roboterchirurgie, Stammzellentherapie, synthetische Biologie, synthetische Genomik, Gewebezüchtung, Tricorder, Gehirn-Computer-Schnittstelle, Neuroinformatik, Elektroenzephalographie, Neuroprothetik, hülsenlose Munition, gerichtete Energiewaffe, Elektro-Laser, elektromagnetische Waffen, elektrothermisch-chemische Technologie, grünes Geschoss, Laser-Waffe, Strahlenwaffe, Schallwaffe, Tarntechnologie, Wirbelringkanone, Elektroschockwaffe, künstliche Schwerkraft, Stasiskammer, aufblasbares Weltraum-Habitat, Miniatursatellit, Android, Nanorobotik, Exoskelett, selbstkonfigurierender Roboter, unbemanntes Fahrzeug, luftlose Reifen, Fahrzeug mit alternativen Kraftstoffen, Elektro-hydrodynamischer Antrieb, Fluidik, Fusionsrakete, Schwebefahrrad, Jetpack, Rucksackhelikopter, Magnetschwebebahn, Vactrain, magnetische Schwebetechnik, Massenantrieb, Personal Rapid Transit, physisches Internet, Roller-Sharing-System, fliegendes Treibstofflager, wiederverwendbares Startsystem, Raumaufzug, Raumflugzeug, Überschalltransport, Fahrzeugkommunikationssystem.
