# Supplementary material for: Innovation indicators based on firm websites—Which website characteristics predict firm-level innovation activity?
Source: PLoS One. 2021 Apr 5;16(4):e0249583. doi: 10.1371/journal.pone.0249583 (PMC8021193; doi:10.1371/journal.pone.0249583)
Supplement: S2 Text — Detailed information on the feature generation. (PDF) [file pone.0249583.s008.pdf]

S2 Text: Detailed information on the calculation of features

Text-based features:

1) **Texts** – To identify the most relevant terms when predicting a firm's innovation status, we transform the scraped texts into a format that allows to do mathematical operations: We convert the website texts into a term-document matrix (e.g., [1], [2]), which is a matrix that counts the frequency of terms that occur in a collection of documents (websites in this particular case). Every column represents a document and a row represents a word from a predefined vocabulary space. Accordingly, every cell counts how often a particular word appears in a particular document. We define our vocabulary space as the 5,000 most frequent words in our entire training text corpus. Before we calculate the term-document matrix, we conduct the following preprocessing steps. First, we merge all scraped subpages related to a single firm and delete irrelevant subpages (imprints, information about cookies or texts that are prescribed by law) by using the gold standard approach based on a supervised machine learning regression model, see [3]. Also, every word is converted into lower case and lemmatized by means of the Python package *spacy*. We exclude punctuation as well as English and German stop words (word lists are derived from the Python package *nltk*). Additionally, we manipulate the term-frequency counts by the TF-IDF scheme [1] as it usually improves predictions. Therefore, each document is tokenized and the term-document frequency is calculated by means of the *TfidfVectorizer* algorithm from *scikit-learn*.

2) **Emerging technology terms** – To capture firms that mention emerging technologies, we conduct a keyword search in which we calculate whether a technology from Wikipedia's list of emerging technologies (Retrieved from https://en.wikipedia.org/wiki/List_of_emerging_technologies accessed on August 16, 2018) appears on a firm's website using all subpages and the entire vocabulary as well as the Python package *regex*. We only search for a selection of technologies that are in a research, development, diffusion or commercialization stage, as it is a criterion for an innovation to be brought into use. A detailed list of all used keywords is provided in S1 Text. The feature *emerging_tech* is a dummy variable that captures whether an emerging technology term appears on a firm website.

3) **Latent patterns** – Latent patterns on a website, which might reveal a firm's innovation status, are captured by the latent Dirichlet allocation model (LDA) (see [2]). The LDA algorithm assumes that a document consists of a set of topics, while every topic is a distribution of words. By linking each word in a document to a topic and iteratively improving assignments, the algorithm learns the distribution of topics in the text corpus as well as the distribution of words related to each topic. Moreover, after applying the LDA algorithm, the topic-document matrix shows how much every topic contributes to a document (website). We do not want our topic model to be exclusively valid for our sample. Hence, we calibrate our topics on a separate sample which consists of 32,276 websites of firms observed in the MUP 2019 but not in the MIP 2019. We apply the same text preprocessing to it as to our MIP sample, with two differences. First, we use a larger vocabulary space (15,000 most frequent words). Second, we do not manipulate word counts by means of the TF-IDF formula, but generate a TF-IDF stop word dictionary excluding words with a lower sum of TF-IDF scores within the LDA corpus than 3. The latter is applied to ensure that rather words that are characteristic for particular websites are considered. Also, to improve our model performance, we delete all words that appear less than 50 times and in more than 90 percent of all documents in the LDA corpus. We use the *TfidfVectorizer* to calculate the stop word dictionary. This dictionary as well as the *CountVectorizer* from *scikit-learn* is applied to generate a term-document matrix for our LDA sample. A term-document matrix for the

MIP sample is calculated in the same manner. The Python package *scikit-learn* is used to train the LDA model. In the standard LDA approach, the number of topics needs to be defined. To solve this issue, we apply the grid-search technique to optimize the number of topics. For this, we use the *GridSearchCV* algorithm from *scikit-learn.* It is evaluated which model parameter combination leads to the best result according to the log likelihood. We conduct a grid-search over different values for the ‘number of topics’-parameter as well as the document-topic prior. We try 200, 180, 150, 250 topics and values of 0.05, 0.1 for the document-topic prior. The optimal number of topics is 150, the highest log-likelihood is achieved with a document-topic prior of 0.1. After fitting the LDA model with the separate sample, the topic distribution for each website in our MIP sample is predicted (*LDA topic*) and used in our Random Forest models, i.e., the predicted topic share in a document for each topic is used as a feature.

4) **Topic popularity index** – The topic popularity index is the sum of document-topic probabilities weighted by the relative frequency each topic appears in the entire text corpus (*pop_score*). A topic is considered to appear in a document if the document-topic probability is larger than 2%.

5) **Language classification** – The export orientation of a website might provide information about a firm’s innovation status. English is worldwide the most widely spoken language by the total number of speakers. Therefore, it is quite likely that firms with international customers describe their products in English. We measure the share of subpages in English language, as well as all other languages except German to approximate the export orientation of a firm (*english_language*, *other_lang*). For the language classification of subpages, we apply the Python package *langdetect.*

6) **Share of numbers** – We also test whether the share of numbers in the total text length per document relates to the innovation status. The share was calculated by the ratio of digits within a string (document). For example, the text ’This book costs 500 dollars.’ has a ratio of 3/28, i.e., 10.7 percent. The corresponding variable is named *share_numbers.*

7) **Flesch-reading-ease score** – The Flesch-Reading-Ease score is a metric used to assess the complexity of texts. The main idea for the index is that short words and short sentences are easier for readers to understand. The Python package *ReadabilityCalculator* (Retrieved from https://pypi.org/project/ReadabilityCalculator/) was used to calculate the score. The full definition can be found in [4] and the corresponding variable is named *flesch_score.*

Meta information features:

8) **Website size** – Approximating firm size might help to predict a firm’s innovation status. For example, [5] show that the number of subpages correlates with firm size and larger firms tend to be more likly to implement an innovation. Hence, we use the number of subpages as a feature to predict a firm’s innovation status (*nr_pages*). One problem related to this feature is that it is truncated at 50 subpages due to the scraping limit of the web-scraper. However, as only 1.5 percent of our observations exceed the scraping limit, we do not see a severe problem here. Moreover, we use a Random Forest model that selects cut-off points for splitting. Hence, it can cope with truncated features. We additionally analyze to what extent the number of characters per website (*text_length*), which might also relate to firm size, informs about the firm’s innovation status.

9) **Loading time** – This feature serves as a proxy for a firm’s hardware structure. A website’s loading time (*load_time*) is determined by a http or https request. The time from sending the request until the arrival of the response is measured. Servers which are far away or which only process the requests slowly (e.g., due to bad hardware or an overload) have a higher loading time (in milliseconds). However, it should be noted that

the IT infrastructure can also be outsourced to professional hosting firms. We retrieved the loading time by means of the the Python packages *requests* and *time*. The latter is a standard Python library.

10) **Mobile version** – For each website, it is retrieved whether a version for mobile end user devices exists. A Google API (https://www.googleapis.com/pagespeedonline/v3beta1/mobileReady?url=http://) is used to extract this information from the websites. The data is delivered as JSON object. Within the delivered data, the binary variable ”score” within the data structure “usability” is used (*mobile_version*). It indicates Google’s mobile version passing score. The Python packages *json*, *mechanize*, *socket* and *urllib* are used for this exercise.

11) **Website age** – To determine the website age, we use web.archive.org. The website includes an Internet archive that allows to look at websites at earlier stages. We wrote a small program that automatically goes to web.archive.org and searches for the first entry of a particular website. This characteristic serves as a proxy for the digital age of a firm (*domain_purchase_year_proxy*). Our program uses the Python package *urllib*.

Network features:

12) **Centrality** – Relationships with other firms might also link to a firm’s innovation status. If a firm is related to another firm, it is likely that the firm will refer on its website to it. Hence, to capture relationships with other firms, the sum of outgoing (*outgoing_links*) and incoming (*incoming_links*) hyperlinks to other firms is observed. Outgoing hyperlinks are measured by the number of external links on a firm website. We measure incoming hyperlinks by counting how often firms which are listed in the entire MUP refer to a particular firm. Additionally, a directed graph is constructed. Here, a vertex represents a firm and an edge a hyperlink from one firm to another. The Pagerank centrality measure is calculated with the Python package *igraph* (https://igraph.org) and the function ”pagerank”. The default parameters are used and the resulting variable is called *pagerank_index*.

13) **Social media** – The use of social media could also be correlated with the firm’s innovation status. Therefore, the sum of hyperlinks to the websites Facebook, Instagram, Twitter, YouTube, Kununu, LinkedIn, XING, GitHub, Flickr, and Vimeo is counted and used as another feature (*social_media*). This is calculated by means of *regex* again.

14) **Bridges** – An undirected graph is constructed, as well. A bridge is an edge of a graph whose removal increases the number of connected components. For each vertex, we count the number of times it is part of a bridge. The Python package *networkx* (https://networkx.github.io) and the function ”bridges” is used to calculate the bridges and the described measure. The resulting variable is named *bridge_index*.

References

[1] Baeza-Yates R, Ribeiro-Neto B, et al. Modern Information Retrieval. vol. 463. New York: ACM press. 1999.

[2] Blei DM, Ng AY, Jordan MI. Latent Dirichlet allocation. Journal of Machine Learning Research. 2003; 3(Jan): 993–1022.

[3] Kinne J, Lenz D. Predicting innovative firms using web mining and deep learning. ZEW Discussion Paper (19-001). [Preprint] 2019 [posted 2019 Jan; revised 2019 Dec; cited 2020 Oct 1]. Available from: http://ftp.zew.de/pub/zew-docs/dp/dp19001.pdf

[4] Flesch R. A new readability yardstick. Journal of Applied Psychology, 1948. 32(3): 221–233.

[5] Kinne J, Axenbeck J. Web mining of firm websites: A framework for web scraping and a pilot study for Germany. Scientometrics. 2020: 1–31.
